# R-Loop Formation in Meiosis: Roles in Meiotic Transcription-Associated DNA Damage

**DOI:** 10.3390/epigenomes6030026

**Published:** 2022-08-24

**Authors:** Yasuhiro Fujiwara, Mary Ann Handel, Yuki Okada

**Affiliations:** 1 Institute for Quantitative Biosciences, The University of Tokyo, Bunkyo-ku, Tokyo 113-8654, Japan; 2 The Jackson Laboratory, Bar Harbor, ME 04609, USA

**Keywords:** DNA damage, homologous recombination, gametogenesis, meiosis, R-loops, spermatogenesis

## Abstract

Meiosis is specialized cell division during gametogenesis that produces genetically unique gametes via homologous recombination. Meiotic homologous recombination entails repairing programmed 200–300 DNA double-strand breaks generated during the early prophase. To avoid interference between meiotic gene transcription and homologous recombination, mammalian meiosis is thought to employ a strategy of exclusively transcribing meiotic or post-meiotic genes before their use. Recent studies have shown that R-loops, three-stranded DNA/RNA hybrid nucleotide structures formed during transcription, play a crucial role in transcription and genome integrity. Although our knowledge about the function of R-loops during meiosis is limited, recent findings in mouse models have suggested that they play crucial roles in meiosis. Given that defective formation of an R-loop can cause abnormal transcription and transcription-coupled DNA damage, the precise regulatory network of R-loops may be essential in vivo for the faithful progression of mammalian meiosis and gametogenesis.

## 1. Introduction

Mammalian spermatogenesis consists of distinct stages of differentiation: spermatogonia, spermatocytes, round spermatids, and elongating/elongated spermatids. Germ cells at each stage exhibit characteristic patterns of transcriptional regulation that trigger the expression of cell type-specific genes, which is critical for precise spermatogenic cell differentiation. Specifically, meiotic transcription occurs in complicated circumstances, as it proceeds in parallel with dynamic genomic changes, including meiotic DNA replication, double-strand breaks, and repair. Knockout of genes involved in these processes frequently results in meiotic defects and pachytene arrest due to the accumulation of DNA damage [1,2,3,4]. Consequently, distinguishing between phenotypes specific to the function of the knocked-out gene and the associated DNA damage is challenging.

An R-loop is a focal, highly ordered three-stranded structure composed of a stable RNA–DNA hybrid and a displaced DNA strand. While DNA–RNA hybrids are physiologically formed in certain regions with a physiological function, R-loops are generally considered pathological and detrimental products that interfere with the transcription process and subsequently contribute to genome instability because unstructured single-stranded DNA is targeted for damage [5]. Among the enzymes involved in R-loop biogenesis, senataxin (SETX) is an R-loop-specific DNA/RNA helicase whose C-terminal SEN1 domain shares a high similarity with yeast Sen1p [6,7,8]. Because Sen1p unwinds the R-loop [9,10,11], the probable mammalian ortholog SETX is believed to have a similar protein function; however, such activity has not yet been reported in vitro. However, *Setx* deficiency does cause the accumulation of DNA damage in somatic cell lines [12]. Notably, SETX is ubiquitously expressed in various cell types, and genetic mutations of it are found in patients with amyotrophic lateral sclerosis 4 (ALS4) [7,13] and ataxia-oculomotor apraxia (AOA2) [14,15]. However, the phenotype of knockout mice is restricted to male infertility due to meiotic arrest [16,17], suggesting that male meiosis is the most susceptible to the effects of R-loop abnormalities in vivo. Consistent with previous studies in somatic cells, *Setx* deficiency caused a massive accumulation of DNA damage in spermatocytes [16,17]. This also raised the question of the extent to which impaired meiotic transcription affects the phenotype.

In the past few years, significant progress has been made in the study of R-loops. They have been implicated in numerous cellular events, such transcription, DNA damage, and DNA replication [5,18,19,20,21,22,23,24,25,26]. However, the physiological role of R-loops in gametogenesis, especially in meiosis, has not been well highlighted, despite the significant phenotype in the knockout mice. Thus, in this review, we will propose mechanisms by which R-loops could contribute to precise meiotic progression during spermatogenesis and point to new directions in research on these structures.

## 2. Transcription and DNA Damage Repair during Meiosis

**Figure 1:**
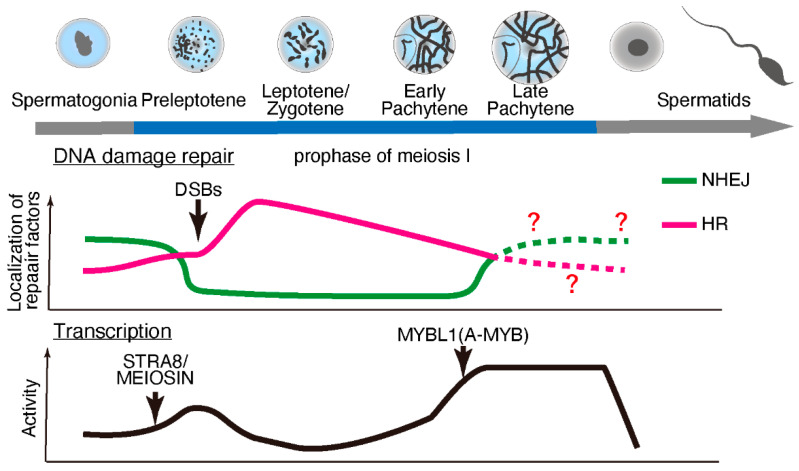
DNA damage repair pathways and transcriptional activity during meiotic progression during spermatogenesis. During early prophase, double-strand breaks (DSBs) are repaired through homologous recombination (HR), and non-homologous end-joining (NHEJ) is suppressed. The localization of HR repair factors decreases, while NHEJ factors start to appear during later prophase. At the onset of meiosis, meiotic gene transcription is activated by STRA8/MEIOSIN, and then transcriptional activity is suppressed until a transcriptional burst is brought about by MYB proto-oncogene like 1 (MYBL1; also known as A-MYB) during the late pachytene stage.

Meiosis is specialized cell division that occurs during gametogenesis that is distinct from the somatic cell cycle. During evolution, meiosis has been a critical driver of genetic diversity in healthy gametes [27]. In mammalian species, meiosis occurs during late embryonic development in females and after birth in males (making it more experimentally accessible in males). Retinoic acid stimulates premeiotic DNA replication and subsequent meiotic gene expression [28,29] through the protein “stimulated by retinoic acid 8” (STRA8) [30] and its interacting partner, MEIOSIN [31]. However, the expression of some meiotic genes, including *Rec8*, is independent of STRA8/MEIOSIN [32,33]. STRA8/MEIOSIN disappears from the nucleus as spermatocytes enter meiotic prophase [31,34,35]. Notably, fewer temporal marker genes are associated with the early prophase stages than with later stages of spermatogenesis [34]. These findings suggest lowered or suppressed transcriptional activity during early prophase stages (until the early pachytene stage), which is supported by earlier studies on the incorporation of radiolabeled uridine or cytidine [36,37] or immunolabeling of RNA Pol II [38]. Subsequently, transcriptional activity becomes exceptionally high during the mid-to-late pachytene stages, regulated in part by MYBL1 [36,37,39,40]. (Figure 1).

Upon meiotic initiation, the meiosis-specific endonuclease SPO11 [41,42] and its interacting partner TOPO6BL [43,44] generate programmed DNA double-strand breaks (DSBs). These DSBs are repaired by meiosis-specific DNA damage response factors through homologous recombination (HR) [45,46,47,48]. In somatic cells, DNA lesions can be repaired through error-prone non-homologous end-joining (NHEJ), which ligates break ends, or error-free HR, a homology-directed repair pathway. In contrast, meiotic cells use HR as the primary repair pathway, especially during early prophase I. Notably, meiotic cells require approximately four days (in male mice) to complete HR during the early meiotic prophase. In addition, meiotic spermatocytes maintain a significantly high transcriptional activity for approximately 9–10 days during the late meiotic prophase. Although it remains unclear why mammalian meiosis requires such an extended period for the late meiotic prophase along with intensive chromosome remodeling, a precise balance between HR and transcription in a stage-specific manner may play a crucial role during meiosis.

Exclusive transcriptional activity coupled with HR in mammalian meiotic cells (Figure 1) requires avoidance of mutations in order to ensure genome integrity. The presence of DNA fragments caused by the double-strand breaks during early meiotic prophase minimizes transcriptional activity because DNA lesions or fragmented DNA slow down Pol II progression [49] or increase the risk of mutations [50]. Furthermore, DNA lesions within genes compromise transcription fidelity via Pol II and promote the persistent formation of R-loops (discussed below) [51,52], thereby increasing the risk of further DNA lesions.

Meiotic DSBs and HR activity are preferentially observed in recombination hotspots, where progenitor cells show a high rate of homologous recombination, activated by the histone methyltransferase, PRDM9 [27]. To avoid the risk of gene expression due to mutations, recombination hotspots are located away from the gene promoter region [53]. It remains unclear whether accidental DSBs generated in non-hotspot regions can be adequately repaired using HR.

## 3. R-Loops and Meiotic Transcription

**Figure 2:**
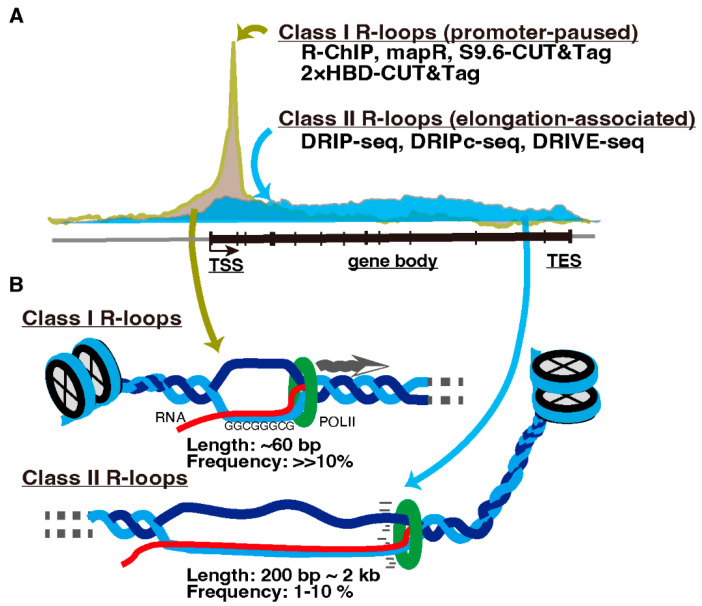
Classes of R-loops. (**A**) R-loops are classified into promoter-paused Class I and elongation-associated Class II R-loops. Methods to detect these R-loops are listed and described in the text. TSS, transcription start site. TES, transcription end site. (**B**) Cartoon illustrating Classes I and II R-loops.

Transcription-associated R-loop formation is proposed to be divided into two classes. One is promoter-paused R-loops (Class I), and the other is elongation-associated R-loops in gene bodies (Class II) (Figure 2, reviewed in [19]). R-loops in each class are preferentially detected by different detection methods. Immunoprecipitation-based protocols, which require nucleic acid extraction and DNA fragmentation prior to antibody application such as DRIP-seq and DRIVE-seq [54], detect R-loops predominantly on the gene bodies (Class II). In contrast, R-loop mapping approaches without nucleic acid extraction such as MapR [55] and S9.6/2 × HBD-CUT&Tag [56], or the R-ChIP method, applying R-loop recognition in cells by adding a catalytically inactive form of RNASEH1 followed by DNA fragmentation and immunoprecipitation [57], detect R-loops primarily in the promoter-proximal regions (Class I) [18,56]. This contradiction appears to be due to the short (approximately 60 bp) and unstable structure of promoter-paused Class I R-loops [58]. Therefore, immunoprecipitation-based methods likely fail to capture them after fragmentation [18,59].

Transcription-associated R-loop structures contain ssDNA susceptible to environmental stress and, thus, could be a source of DNA damage. Therefore, an R-loop must be resolved immediately by R-loop-specific ribonucleases RNaseH1/RNaseH2 and/or the DNA/RNA helicase SETX in collaboration with other R-loop-associated factors. Failure of R-loop resolution due to the defects of these molecules can compromise DNA repair in somatic [60,61,62,63,64] and meiotic cells [16,17]. Proper regulation of R-loop formation in germline cells safeguards genome integrity, especially during the late meiotic prophase when transcription activity is exceptionally high [36,37,39].

Structural analyses have attempted to determine the mechanism by which R-loop-binding proteins recognize DNA/RNA hybrids. Unlike mammalian RNASEH1, which recognizes DNA/RNA hybrids as a single molecule [65,66], RNASEH2 functions as a complex containing three subunits (RNASEH2A, RNASEH2B, and RNASEH2C) and recognizes DNA/RNA hybrids [67]. Although the mechanism by which these RNaseH proteins selectively recognize DNA/RNA hybrids or R-loops was demonstrated [66,68,69,70], the functions of RNaseH1 and RNaseH2 are differentially regulated through the cell cycle [71]. In contrast, DNA/RNA helicases, SETX, and yeast SETX ortholog sen1 contain two nucleotide-binding RecA domains within their helicase domains, and each domain recognizes DNA or RNA [11].

**Figure 3:**
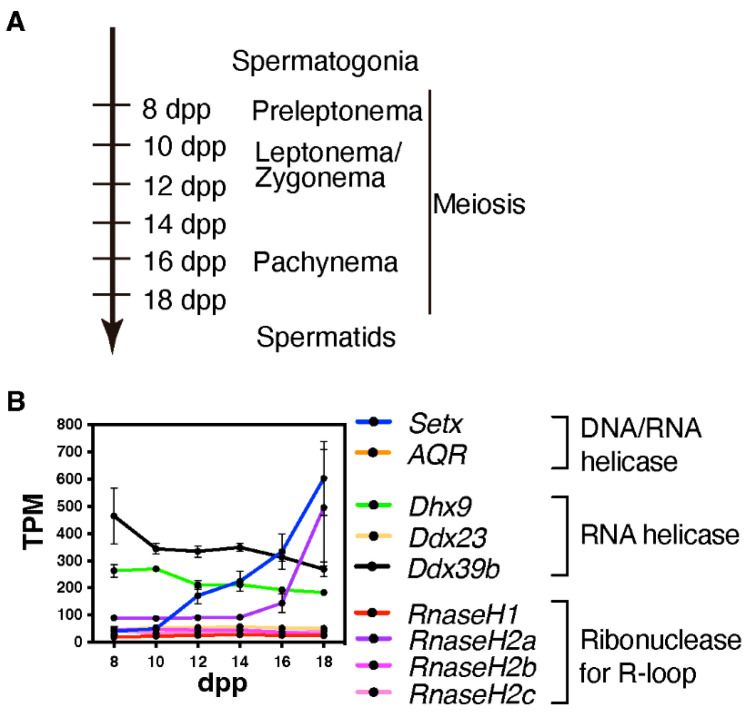
Expression of R-loop-associated genes during the first wave of spermatogenesis. (**A**) This time-line indicates the predominant cell types in the testis at points during the first wave of spermatogenesis; dpp = days post partum (**B**) Line plot depicting the developmental pattern of the expression of R-loop-associated genes during the first wave of spermatogenesis; TPM = transcripts per million. Publicly available RNA-seq data were used to generate this plot (GSE72833) [34].

We currently lack studies in mice or other mammalian model that elucidate whether and where DNA/RNA hybrids or R-loops are formed during mammalian meiosis and the possible sex-specificity. However, given that several R-loop formation-associated genes, specifically *Setx* and *RnaseH2a*, are highly expressed during the late prophase in spermatogenesis (Figure 3), these genes might play a role in the formation of R-loop or DNA/RNA hybrids in a stage-specific manner.

A study on a homology search in a yeast model sheds light on the function of DNA/RNA hybrids during meiosis. In meiosis, single-end invasion by ssDNA, which occurs during strand exchange between a resected DSB end and its homolog, plays a crucial role in homology searches during meiosis [72]. DNA/RNA hybrids are formed by annealing RNA fragments to the resected ssDNA ends. These DNA/RNA hybrids mediate homology searches via RAD52 [73]. Similar functions have been reported for DNA/RNA hybrids in somatic cells [74,75,76,77,78,79,80,81,82]. Given that DEAD-box RNA helicases, *Dhx9* and *Ddx39b*, both of which are implicated in various type of cancers [83,84], exhibit relatively high expression levels during early meiotic prophase (Figure 3), these factors might function in mammalian meiotic HR by recognizing DSB-associated DNA/RNA hybrids. As resected ssDNA extends approximately 2 kb from DSB sites in mice [85], DNA/RNA hybrids that are likely formed during mammalian meiotic HR are expected to reach up to hundreds of bp to 1 kb, which is longer than promoter-associated Class I R-loops containing the region with DNA/RNA hybrids over approximately 60 bp and transcriptional elongation-associated Class II R-loops (approximately 300 bp up to several kb) [18,58]. Further epigenomic analyses revealed that DNA/RNA hybrids or R-loops are associated with meiotic HR.

## 4. Physiological Functions of R-Loops and Their Regulating Factors: Transcription Regulator or Harmful Source of DNA Damage

As R-loops play a crucial role in vivo [5,20,51], elucidating the functions of meiotic R-loops or DNA/RNA hybrids during meiosis is crucial. Although RNaseH1-deficient mice [86] or mice with mutated RNaseH2 subunit-encoding genes [87,88,89] exhibit perinatal or embryonic lethality, mutations in *RNASEH2* [90,91] and *SETX* genes [13,92] in humans are known to cause neurological disorders, indicating vital organ-specific physiological roles. Furthermore, *Setx* is required for successful meiosis in male mice [16,17] and probably in humans [93,94]. *Setx*-deficient mice exhibited unrepaired DSBs in the autosomes of meiotic spermatocytes at the pachytene stage, at a time when autosomal DSBs are repaired in wild-type pachytene spermatocytes. Because *Setx*-deficient spermatocytes exhibit unresolved R-loops, the aberrant DSBs found in *Setx*-deficient spermatocytes are probably derived from unresolved R-loops or aberrant DNA damage repair [16,17]. Furthermore, *Setx*-deficient mice exhibit defective formation of the sex body, a chromatin domain in which the entire X and Y chromosomes are silenced [95,96,97,98], indicating that SETX and R-loops play a role in non-canonical transcriptional regulation, such as meiotic sex chromosome inactivation [99].

Analyses of *Setx*-deficient mice have also suggested a direct association between SETX and HR. *Setx*-deficient spermatocytes showed an aberrant increase in the number of DNA damage repair-related markers, including RAD51, DMC1, and SPATA22, and the loss of MLH1, a marker for crossover [16,17]. In human-cultured cells, SETX interacts with breast cancer type 1 susceptibility protein (BRCA1) [12]. Although BRCA1 is not essential for meiotic HR, BRCA1 seems to play a role in the timing of crossover formation [100]. In somatic cells, BRCA1 is required to recruit SETX to DNA damage sites [12], indicating a similar function for BRCA1 in meiotic DNA damage repair. SETX also interacts with the DNA-activated protein kinase catalytic subunit (DNA-PKcs) [101] that activates NHEJ [102]. Taken together, SETX might play a role in the later steps of HR, especially crossing over and DNA damage repair during the late prophase. It may also be that the DNA damage and residual DNA damage repair proteins observed in *Setx*-deficient pachytene spermatocytes are a consequence of inefficient homology searches via DNA/RNA hybrids at the DSB sites. In these ways, the *Setx*-mutant mice may provide unexpected insights into the formation and function of R-loops and any possible sexual dimorphism.

## 5. R-Loop Formation in Meiotic Genes

R-loops are formed primarily on the GC-rich sequences [103,104], although R-loop-binding factors, such as RNASEH1, do not exhibit binding bias toward GC-rich sequences [56]. High C/G sequences are notably found near the promoter region, the so-called CpG islands, where methylation of cytosines (commonly known as DNA methylation) suppresses transcription [105]. Promoters of meiotic genes contain CpG islands that are methylated before meiosis. These methylated promoters are demethylated by TET1 upon meiosis initiation in female meiosis [106], although no study has shown TET1-dependent demethylation of promoter-proximal CpG islands in male meiosis. Repeated formation of R-loops during the pachytene stage could possibly mediate prolonged robust gene expression by suppressing de novo DNA methylation [54]. Because yeast Sen1P and its mammalian ortholog SETX (in cultured somatic cells) reportedly interact with RNA polymerase II [107,108], Sen1P/SETX is likely directly involved in transcriptional activity. Furthermore, R-loops associated with SETX and/or RNASEH2A could promote or maintain the recruitment of transcription factors to gene promoter regions in association with the open DNA structure that is characteristic of the pachytene stage. Further studies using mouse models should focus on the meiosis-specific functions of R-loops and their associated factors, as well as the germline-specific R-loop regulatory network during meiosis.

**Figure 4:**
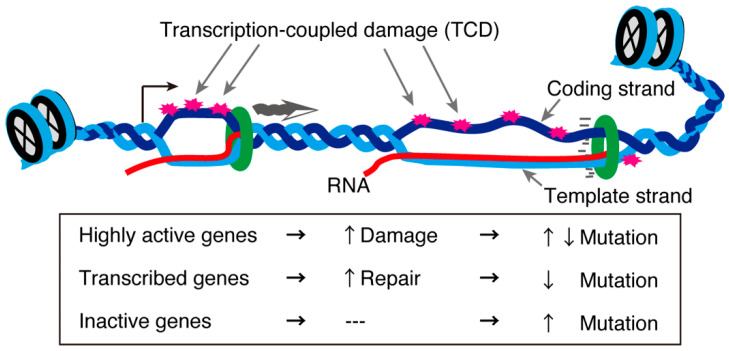
Transcription-coupled DNA damage and mutation rates. Unwound DNA structures during transcription are susceptible to environmental stress, creating transcription-coupled damage (TCD). TCDs in genes with a normal transcription level are repaired through transcription-coupled repair mechanisms, while some TCDs in highly transcribed genes or inactive are not repaired, increasing their mutation rates [109].

In the testis, approximately 90% of protein-coding genes are transcribed [109], although most are not actually “used” in terms of protein production during spermatogenesis. Of all the testicular cells, meiotic cells display exceptionally high transcriptional activity [36,37]. Generally, highly transcribed genes are subjected to transcription-coupled DNA damage. This concept was tested in an interesting recent study that analyzed the genome sequences of mice and humans in the context of single-cell transcriptomes of testes. The study revealed that highly transcribed spermatogenic and meiotic genes exhibited a low mutation rate in the human population. However, other highly and lowly transcribed genes showed an increased mutation rate on the single-stranded coding strand [109]. In contrast, moderately expressed genes displayed fewer mutations (Figure 4). These gene in which mutations were detected were biased towards those involved in the immune response [109]. These asymmetrical mutations in the testis indicate a gene- or strand-specific repair machinery probably associated with R-loops. Further analyses are required to clarify the meiosis-specific transcription-coupled DNA damage repair machinery and how it might be related to the meiotic requirement for R-loop-associated proteins.

## 6. Concluding Remarks: Precise Regulation for R-Loops and Catastrophic DNA Damage during Meiosis

Some meiotic genes, including *Spo11* and *Rnf212*, function in a dose-dependent manner [110,111,112,113]. Therefore, the precise regulation of transcription is essential during early prophase, especially at the onset of meiosis, by both STRA8/MEIOSIN-dependent regulation [30,31] and STRA8-independent regulation. In contrast, during the pachytene stage, robust expression of pachytene genes plays a crucial role in spermiogenesis and embryo viability [34]. Transcript levels may be regulated during subsequent stages, probably in part by pachytene piRNAs [114,115,116]. Because pachytene gene transcription results in a large and diverse transcriptome, the mode of R-loop regulation by RNaseH, which degrades RNA, should be suppressed. Therefore, unlike canonical R-loop regulation during the somatic cell cycle, the meiosis-specific mode of R-loop regulation might depend solely on a helicase-mediated unwinding mechanism by SETX. However, the mechanism by which the activity of R-loop regulation is controlled remains unclear; this is important to resolve because either too many or too few R-loops pose a risk to genome integrity and sufficient levels of transcripts during the pachytene stage. Furthermore, the mechanism by which the two modes of R-loop regulation, degradation and unwinding modes, cooperate or are selectively suppressed in both somatic and meiotic cells remains unclear. Elucidating the properties and cell cycle- or cell-type specificities of R-loops detected by each regulatory factor is essential. Furthermore, clarification of the putative role of DNA/RNA hybrids in mammalian meiotic HR will generate new insights into the still imperfectly understood mechanisms that drive this essential aspect of meiosis.

In conclusion, mammalian meiotic germ cells, at least in the male, appear to deploy a more complex R-loop regulatory network than somatic cells. This requires precise regulation to secure both the robust gene expression that supports gamete differentiation and the genome integrity that ensures continuity of germline cells. Recent studies reviewed here [18,56,57,59,103,117,118] demonstrate that R-loop detection is now experimentally feasible and should play a central role in the study of mammalian meiosis.
